# Clinical and Imaging Data-based Machine Learning for Early Diagnosis of Bronchopulmonary Dysplasia: A Meta-analysis

**DOI:** 10.2174/0115734056421036250806053617

**Published:** 2025-08-08

**Authors:** Yilin Chen, Huixu Ma, Xi Liu

**Affiliations:** 1 Department of Thoracic Surgery, Chongqing General Hospital, Chongqing University, Chongqing 401147, China; 2 Department of Trauma Orthopaedics, Chongqing General Hospital, Chongqing University, Chongqing 401147, China; 3 Department of Radiology, Chongqing Hospital of Traditional Chinese Medicine, Chongqing 400021, China

**Keywords:** Bronchopulmonary dysplasia, Clinical and imaging data, Machine learning, Meta-analysis, Validation strategies, VLBW, ELBW

## Abstract

**Introduction:**

This meta-analysis aimed to evaluate the diagnostic performance of Machine Learning (ML) models for early prediction of bronchopulmonary dysplasia (BPD) in preterm infants, addressing the need for timely risk stratification.

**Methods:**

Systematic searches of PubMed, Embase, and other databases identified 9 eligible studies (12,755 infants). Data were extracted and pooled using bivariate generalized linear mixed models. Study quality was assessed *via* QUADAS-2.

**Results:**

ML models demonstrated high accuracy (pooled sensitivity: 0.81, specificity: 0.85, AUC: 0.90). Multimodal models and ensemble algorithms (*e.g*., Random Forest) outperformed single-modality approaches. Models using data from the first 7 postnatal days achieved superior performance compared to those using data from day 28.

**Discussion:**

ML enables ultra-early BPD prediction, preceding conventional diagnosis by weeks. Heterogeneity in data modalities and validation strategies highlights the need for standardized reporting.

**Conclusion:**

ML-based BPD prediction shows promise for clinical translation but requires prospective validation and cost-effectiveness analysis.

## 1 INTRODUCTION

Bronchopulmonary dysplasia (BPD), the predominant chronic respiratory morbidity affecting preterm neonates, especially those with Very Low Birth Weight (VLBW) or Extremely Low Birth Weight (ELBW) [1, 2], exhibits a rising occurrence rate concomitant with progress in neonatal intensive care [3, 4]. Among infants born before 28 weeks' gestation, BPD prevalence can reach 50%, and ranges from 19.3% to 85% in those weighing under 1000 grams at birth [4]. This chronic pulmonary disorder not only elevates neonatal mortality risk but is also strongly linked to significant long-term morbidities, such as neurodevelopmental deficits, repeated respiratory infections, and impaired growth [5, 6], resulting in considerable burdens for families and society.

The conceptualization of BPD has shifted from “classic” to “new” forms. Initially delineated by Northway in 1967, classic BPD primarily affected preterm infants necessitating high-concentration oxygen and mechanical ventilation for severe respiratory distress syndrome, who remained oxygen-dependent at 28 postnatal days [7]. Moreover, the understanding of pediatric respiratory diseases has evolved in tandem with BPD, with a growing recognition of the complex interactions between environmental exposures and early-life pulmonary development. This broader respiratory health context underlines the urgency of early and accurate prediction models to mitigate long-term pulmonary morbidity in vulnerable infants. Advances in perinatal care, including widespread antenatal corticosteroid and postnatal surfactant use, have reduced classic BPD incidence, leading to the emergence of “new BPD”. This entity predominantly manifests in infants ≤1kg, who may present initially with minimal or no lung pathology but develop persistent oxygen requirements extending beyond 36 weeks postmenstrual age (PMA) [1]. The current diagnostic framework, established by the NICHD in 2000, defines BPD as oxygen dependency (FiO_2_ > 21%) lasting >28 days, with severity graded at 36 weeks PMA: mild (room air), moderate (FiO_2_ < 0.30), or severe (FiO_2_ ≥ 0.30 or requiring positive pressure support) [8].

BPD pathogenesis is multifactorial, involving interactions among genetic predisposition, pulmonary immaturity, oxygen toxicity, ventilator-induced damage, infection, and inflammatory cascades [1, 9, 10]. Genetic influences are estimated to contribute substantially (53%-79%) to BPD susceptibility [11], although definitive causative genes remain elusive [1]. The inherent pulmonary immaturity of preterm infants constitutes the foundational vulnerability; lower gestational age corresponds directly with reduced lung maturity and heightened risk of exposure to detrimental factors like hyperoxia, mechanical ventilation, and infection [1]. Critically, inflammation is central to the development of BPD; intrauterine infection or chorioamnionitis exposes the fetus to an inflammatory environment, precipitating massive cytokine release that disrupts lung development and inflicts injury [12].

Machine Learning (ML) technology has recently shown considerable promise for medical predictive modeling. ML algorithms offer advantages over traditional statistical methods by efficiently managing high-dimensional data with non-linear relationships, integrating multi-source heterogeneous variables (*e.g*., clinical data, imaging, biomarkers), and capturing dynamic temporal patterns [13]. Within BPD prediction, several investigations have employed ML models for early risk assessment [14-22]. However, significant heterogeneity exists in study methodologies, predictor selection, and performance evaluation metrics, underscoring the necessity for a systematic appraisal. Recently, ML technology has shown considerable promise. As the first meta-analysis dedicated to ML-based BPD prediction models, this study uniquely synthesizes evidence on multimodal data integration (including clinical, imaging, and genomic biomarkers), explainability techniques (*e.g*., SHAP values), and validation strategy comparisons, moving beyond simple performance aggregation to inform future model development and clinical adoption. This systematic review and meta-analysis aims to comprehensively evaluate the performance characteristics of ML models for early BPD prediction, identify key determinants of model efficacy, and furnish an evidence base to guide future research and clinical implementation.

## 2 METHODS

### 2.1 Literature Retrieval and Screening Strategies

Adhering to the Preferred Reporting Items for Systematic reviews and Meta-Analyses (PRISMA) framework, we conducted systematic literature searches across PubMed, Embase, Web of Science, Scopus, Cochrane Library, and IEEE Xplore databases, covering the period from the inception of each database through April 2025. These databases were selected for their comprehensive coverage of biomedical literature (PubMed/Embase/Cochrane), interdisciplinary research (Web of Science/Scopus), and technical advancements in ML applications (IEEE Xplore). A comprehensive search syntax incorporating both controlled vocabulary (*e.g*., MeSH, Emtree) and natural language terms was employed. Key search concepts encompassed: “bronchopulmonary dysplasia” OR “BPD”, combined with “machine learning” OR “deep learning” OR “artificial intelligence”, AND “prediction model” OR “risk stratification” OR “prognostic model” OR “neonatal chronic lung disease”. Additionally, bibliographies of relevant articles were manually scrutinized to identify additional eligible publications. Please refer to Appendix 1 for the complete search strategy used for PubMed.

Although sources of grey literature (*e.g*., OpenGrey, ProQuest Dissertations) were considered, preliminary searches yielded no eligible unpublished studies meeting our inclusion criteria. Thus, only peer-reviewed publications were included.

Screening was executed in two sequential phases. Initially, two reviewers independently evaluated titles and abstracts to exclude records that unequivocally failed the inclusion criteria. Subsequently, full texts of potentially relevant articles were obtained and meticulously assessed. Disagreements were resolved *via* consensus discussion or adjudication by a third reviewer. Studies were included based on: (1) Population: Preterm infants (<37 weeks gestation); (2) Objective: Development or validation of an early BPD prediction model; (3) Methodology: Utilization of ≥1 machine learning algorithm; (4) Outcomes: Reporting of model performance metrics (*e.g*., sensitivity, specificity, AUC); (5) Accessibility: Full text available. Exclusion criteria comprised: (1) Reviews, editorials, or conference abstracts; (2) Studies lacking original performance data or insufficient metrics for extraction; (3) Non-English publications; (4) Studies relying solely on conventional statistical techniques (*e.g*., Logistic regression) without machine learning application.

### 2.2 Data Extraction and Quality Evaluation

A standardized data extraction form was designed to collect the following information: (1) Basic study characteristics: first author, publication year, study design, country, sample size, number of BPD cases, gestational age range; (2) Model characteristics: prediction time point, type of machine learning algorithm, feature selection method, categories of input variables (*e.g*., clinical variables, imaging data, biomarkers); (3) Model performance: sensitivity, specificity, AUC, True Positive (TP), False Positive (FP), False Negative (FN), True Negative (TN) values; (4) Validation strategy: internal validation (*e.g*., cross-validation, bootstrapping), external validation, prospective validation.

The methodological rigor of the included studies was independently appraised by two reviewers using the Quality Assessment of Diagnostic Accuracy Studies-2 (QUADAS-2) instrument [23]. This tool evaluates risk of bias across four key domains: patient selection, index test, reference standard, and flow/timing. Applicability concerns within each domain were also assessed. Discrepancies in quality ratings were reconciled through consensus discussion, involving a third reviewer if necessary.

### 2.3 Statistical Analysis

Data synthesis was conducted utilizing Stata software (Version 19; StataCorp LP, College Station, TX). This platform facilitated figure generation and computation of pooled estimates for: sensitivity (SEN), specificity (SPE), positive likelihood ratio (LR+), negative likelihood ratio (LR-), Diagnostic Odds Ratio (DOR) (all with 95% confidence intervals, CI), the Summary Receiver Operating Characteristic (SROC) curve, and its Area Under the Curve (AUC) for machine learning models predicting BPD based on clinical and/or imaging data. Pooling of SEN and SPE employed a bivariate generalized linear mixed model.

Between-study heterogeneity was quantified *via* the chi-square test, Cochran's Q statistic, and the Higgins I^2^ statistic. Substantial heterogeneity was inferred if I^2^ > 50%. In cases of significant heterogeneity, potential sources were explored using subgroup analyses and meta-regression.

Publication bias across the body of evidence was evaluated using Deeks' funnel plot asymmetry test, with a significance threshold of *p* < 0.01 indicating potential bias.

## 3 RESULTS

### 3.1 Literature Search Results

**Fig. (1):**
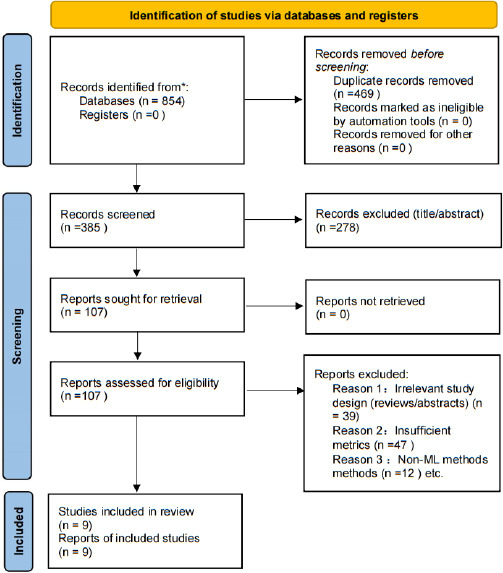
Flow diagram of study inclusion and exclusion.

Our systematic database search initially identified 854 articles. After removal of 469 duplicate records, 278 studies were excluded following title and abstract screening, and a further 107 studies were excluded after full-text review. Consequently, nine studies were included in the final quantitative synthesis (Fig. **1**).

### 3.2 Characteristics of the Included Studies

Nine studies, encompassing a total of 12,755 preterm infants, were ultimately included. Their basic characteristics are detailed in Table **1**. Publication years ranged from 2021 to 2025, reflecting recent advancements in this field. The predominant study design was retrospective cohort (6 studies, 66.7%), followed by prospective cohort (2 studies) and bioinformatics cohort (2 studies). Geographically, the studies were widely distributed, encompassing China, Italy, Denmark, the United States, Canada, and a multinational collaborative study, indicating a global research interest.

Sample sizes ranged from 61 to 9,006 infants, with a mean sample size of 1,395 infants. The reported prevalence of BPD varied substantially, ranging from 12.3% (Montagna 2024 [15]) to 62.0% (Luo 2024 [22]), highlighting the heterogeneity of the study populations. Prediction time points demonstrated an early focus, ranging from birth (Verder 2021 [14]) to postnatal day 28. Notably, four studies (Verder 2021 [14]; Chou 2024 [16]; Khurshid 2021 [19]; Dai 2021 [21]) specifically focused on ultra-early prediction within the first 7 days of life, providing a valuable time window for early clinical intervention.

The included studies employed diverse machine learning algorithms (Table **2**). Ensemble learning methods (Random Forest, XGBoost) were the most prevalent (5 studies, 55.6%), followed by deep learning models (U-Net + ResNet in Chou 2024 [16]) and regularized regression (LASSO, penalized logistic regression). Regarding feature engineering, four studies (44.4%) utilized advanced feature selection methods (*e.g*., Boruta, SVM-RFE, recursive feature elimination) to optimize input variables and mitigate the risk of the curse of dimensionality. In terms of algorithm performance, Random Forest (AUC 0.92, reported by Lei *et al*. [17]) and XGBoost (AUC 0.92, reported by Montagna *et al*. [15]) generally demonstrated superior performance in studies that primarily used clinical variables. Conversely, deep learning models (*e.g*., U-Net+ResNet in Chou *et al*. [16]) showed unique advantages in parsing imaging features.

A trend towards multimodal data fusion was evident, with input variables categorized into three main types: (1) Clinical variables: Gestational age, birth weight, type of respiratory support, duration of mechanical ventilation (included in all studies); (2) Imaging features: Chou 2024 [16] utilized U-Net to segment lung regions on chest X-rays and extract texture features; (3) Biomarkers: Gastric aspirate FTIR spectroscopy (Verder 2021 [14]), gene expression markers (*e.g*., MMP9, Siglec-5, CYYR1) (Zhang 2025 [20]; Luo 2024 [22]), and immune microenvironment characteristics (*e.g*., neutrophil and dendritic cell infiltration) (Zhang 2025 [20]). Notably, Montagna (2024) [15] and Leigh (2022) [18] introduced prenatal factors (Absent/Reversed End-Diastolic Flow in the umbilical artery - AREDF) and dynamic respiratory trajectories (evolution of respiratory support patterns over the first 14 postnatal days), respectively, significantly enhancing the models' temporal predictive capabilities. The Random Forest model developed by Lei (2021) [17], which integrates both static and dynamic factors (similar to Leigh 2022 [18]), achieved the highest performance (Sensitivity = 0.81, Specificity = 0.90, AUC = 0.92).

Regarding model interpretability, Montagna (2024) [15] and Leigh (2022) [18] employed SHAP (SHapley Additive exPlanations) values. Five of the nine studies (*e.g*., Verder 2021 [14]; Lei 2021 [17]; Khurshid 2021 [19]; Dai 2021 [21]) utilized methods such as regression coefficients, feature importance scores, or class activation mapping to identify core predictive factors (*e.g*., birth weight, gestational age, duration of mechanical ventilation), thereby enhancing the models' clinical credibility.

### 3.3 Quality Assessment of Included Studies

The methodological quality of the included studies, assessed using the QUADAS-2 tool [23], is detailed in Table **3**. This evaluation encompassed risk of bias and applicability concerns across four domains: patient selection, index test, reference standard, and flow/timing. The majority of studies exhibited low risk of bias in patient selection, index test, and flow/timing. However, the reference standard domain displayed a higher proportion of studies with unclear risk.

### 3.4 Primary Findings

**Fig. (2):**
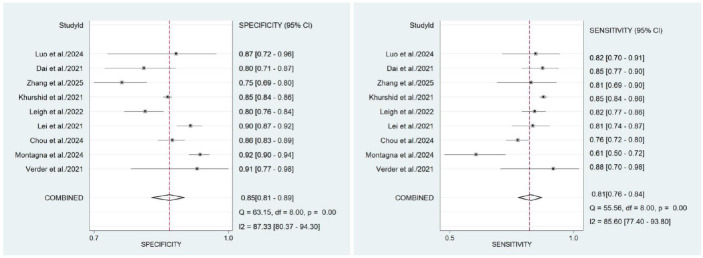
Forest plot of ML model accuracy for BPD prediction. Note: Heterogeneity was quantified *via* I^2^ statistics: <25% negligible, 25–50% low, 50–75% moderate, >75% substantial.

**Fig. (3):**
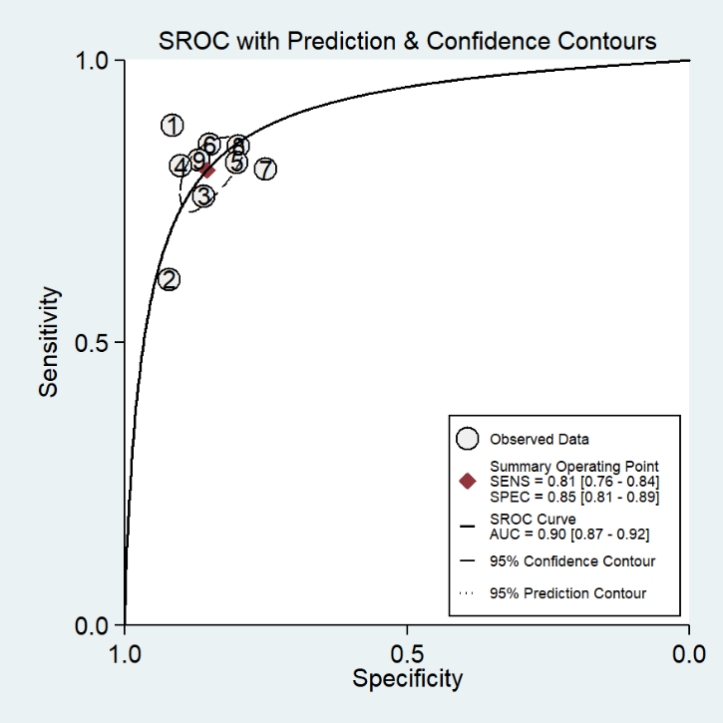
Hierarchical summary receiver operating characteristic (HSROC) curve for ML-based BPD prediction. **Note:** The ellipse represents the 95% confidence region for the summary estimate. Study identifiers: 1= Verder *et al*. [14], 2=Montagna *et al*. [15], 3=Chou *et al*. [16], 4=Lei *et al*. [17], 5=Leigh *et al*. [18], 6=Khurshid *et al*. [19], 7=Zhang *et al*. [20], 8= Dai *et al*. [21], 9=Luo *et al*. [22].

Our meta-analysis demonstrates the robust diagnostic efficacy of machine learning models that utilize clinical and/or imaging data for the early prediction of BPD. The pooled sensitivity was 0.81 (95% CI: 0.76–0.92), and the pooled specificity was 0.85 (95% CI: 0.81–0.89) (Fig. **2**). The Summary Receiver Operating Characteristic (SROC) curve yielded an AUC of 0.90 (95% CI: 0.87–0.92) (Fig. **3**), indicating an outstanding overall discriminative capacity (an AUC approaching 1 signifies superior accuracy).

**Fig. (4):**
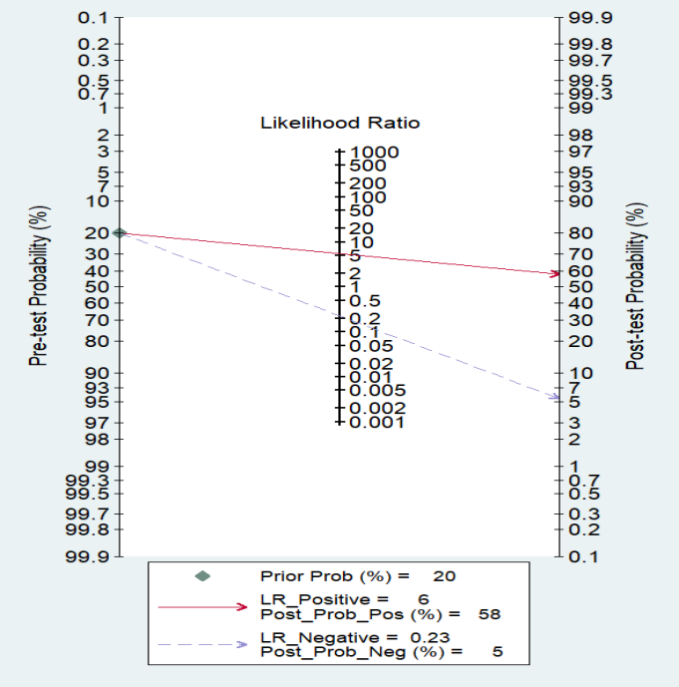
Fagan nomogram illustrating post-test probability of BPD using ML prediction models.

Further metrics revealed a positive likelihood ratio (LR+) of 6.0, indicating that a positive test result significantly increases the probability of a subsequent BPD diagnosis. Conversely, the negative likelihood ratio (LR-) was 0.23, underscoring the model's high proficiency in ruling out BPD (Fig. **4**). Collectively, these findings substantiate the considerable clinical value of these ML models for both confirming and excluding BPD at an early stage.

### 3.5 Evaluation of Publication Bias

**Fig. (5):**
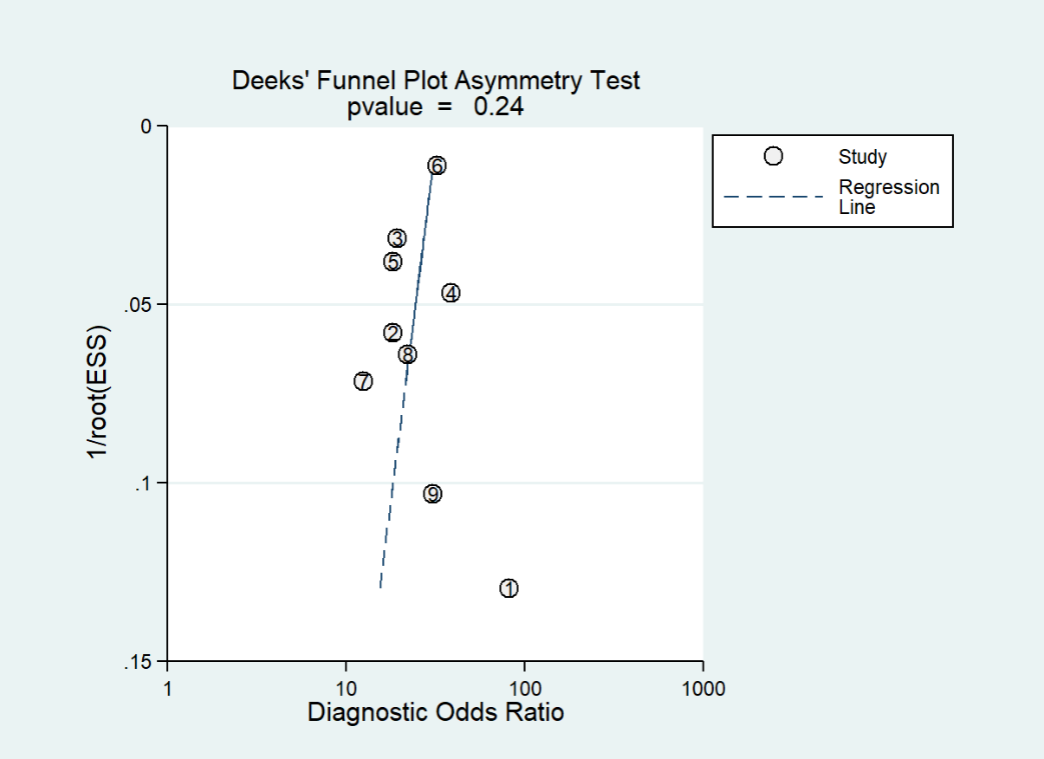
Deeks' funnel plot assessing potential publication bias. **Note:** ESS, effective sample sizes. Study identifiers: 1= Verder *et al*. [14], 2=Montagna *et al*. [15], 3=Chou *et al*. [16], 4=Lei *et al*. [17], 5=Leigh *et al*. [18], 6=Khurshid *et al*. [19], 7=Zhang *et al*. [20], 8= Dai *et al*. [21], 9=Luo *et al*. [22]

Deeks’ funnel plot asymmetry test (t = -1.54; p = 0.24) (Fig. **5**) was employed to assess publication bias. The non-significant result (*p* > 0.01) and visual inspection of the funnel plot provided no substantial evidence for the presence of publication bias.

### 3.6 Heterogeneity Exploration and Meta-Regression

Significant statistical heterogeneity was detected among the included studies (I^2^ = 78.3%, *p* < 0.001). To elucidate potential sources, subgroup analyses and meta-regression were conducted (Table **4**). Meta-regression identified data modality (*p* = 0.04), algorithm type (*p* = 0.02), validation approach (*p* = 0.02), and prediction time point (*p* = 0.01) as significant contributors to heterogeneity. Key subgroup findings included: (1) Data modality: Models integrating multimodal inputs (*e.g*., clinical data + imaging + biomarkers) achieved significantly higher sensitivity and specificity than models relying on a single data source (*p* < 0.05). (2) Algorithm type: Ensemble learning methods (*e.g*., Random Forest, XGBoost) significantly outperformed single-algorithm models in diagnostic performance (*p* < 0.05). (3) Prediction time point: Models utilizing data from the first postnatal week demonstrated significantly enhanced sensitivity and specificity compared to those predicting at 28 days (*p* < 0.05). This capability substantially precedes the standard clinical diagnostic timeframe (28 days), affirming the feasibility of ML for ultra-early BPD risk stratification. (4) Validation strategy: Models subjected to external validation exhibited comparatively lower sensitivity and specificity than those validated only internally, suggesting potential overfitting in internally validated models. Sensitivity analysis excluding the two bioinformatics-only studies [20, 22] yielded a consistent pooled AUC of 0.89 (95% CI: 0.86-0.92). The pooled specificity was higher in studies using the NICHD criteria (0.87 *vs*. 0.82), while sensitivity remained relatively unchanged (0.80 *vs*. 0.81), indicating some robustness to diagnostic heterogeneity (Table **S1**).

## 4 DISCUSSION

This meta-analysis represents the first systematic evaluation of the comprehensive performance of ML models for the early prediction of BPD, synthesizing data from 9 studies encompassing 12,755 preterm infants. Our meta-analysis revealed that machine learning models demonstrated high diagnostic accuracy for early BPD prediction, with a pooled sensitivity of 0.81 and specificity of 0.85. This suggests that ML algorithms are capable of effectively identifying infants at high risk of BPD even before the conventional clinical diagnostic window. The high area under the curve (AUC = 0.90) further confirms their strong overall discriminative capacity. A particularly important finding is that models using data from within the first 7 postnatal days performed significantly better than those using data from postnatal day 28. This indicates that clinically relevant signals for BPD risk can be detected very early in life, which is critical because interventions such as lung-protective ventilation or anti-inflammatory therapy are more effective when implemented in the early stages of lung development.

The superior performance of multimodal models-those integrating clinical data, imaging features, and biomarkers-demonstrates the benefit of capturing the multifactorial nature of BPD pathogenesis. BPD results from a complex interplay of prematurity, mechanical injury, inflammation, and genetic susceptibility. Single-source models may only partially represent these mechanisms, whereas multimodal integration enhances prediction by capturing synergistic effects. Moreover, ensemble learning algorithms (*e.g*., Random Forest, XGBoost) consistently outperformed single-model approaches. These algorithms are better equipped to handle high-dimensional, non-linear, and heterogeneous data-common characteristics of neonatal clinical datasets. Their robust internal validation strategies and resistance to overfitting also contribute to their reliability. Collectively, these findings highlight the potential of ML models to transform BPD risk assessment from a reactive diagnosis to a proactive prevention approach, enabling neonatologists to tailor early interventions based on individualized risk profiles. ML models demonstrated superior accuracy (AUC=0.90) compared to traditional clinical scoring systems (*e.g*., NIH consensus model AUC≈0.75 [24]), though prospective validation of clinical utility remains essential. These results signify the potential of ML technology to provide a critical time window for early intervention. By fusing multi-source heterogeneous variables, such as gestational age, chest X-ray texture features, and genetic biomarkers, ML models enable ultra-early risk stratification within the first 7 postnatal days. This represents an advance of at least 21 days compared to the conventional diagnostic window (36 weeks postmenstrual age), providing a critical time window for implementing targeted interventions (*e.g*., personalized respiratory support, anti-inflammatory therapy) to improve patient outcomes.

The pathogenesis of BPD inherently involves complex, nonlinear interactions among genetic susceptibility, pulmonary immaturity, oxygen toxicity, and inflammatory injury [1, 9, 10]. The strength of the multimodal data fusion strategy lies in its profound alignment with this intricate mechanism. Models relying on a single data source (*e.g*., solely clinical variables or imaging features) can only capture partial pathological processes (*e.g*., ventilator-induced injury or structural abnormalities). In contrast, integrating multimodal data—clinical, imaging, and biomarkers—allows for the parsing of nonlinear synergistic effects between variables. For instance, Chou *et al*. [16] elevated the prediction AUC to 0.87 by combining texture features extracted from U-Net-segmented lung regions on chest X-rays with clinical ventilation parameters. Zhang *et al*. [20] achieved an AUC of 0.81 by integrating neutrophil infiltration characteristics with Siglec-5 gene expression. This “mechanism-driven modeling” strategy not only significantly enhances discriminatory performance but also provides a potential biological rationale for personalized interventions. At the algorithmic level, ensemble learning methods (*e.g*., Random Forest, XGBoost) demonstrated significant superiority. This advantage stems primarily from two factors: (1) inherent resistance to overfitting (*e.g*., Random Forest reduces variance through bootstrap sampling and feature randomness) [25-28], effectively mitigating the common risk of overfitting in medical studies with limited sample sizes; and (2) robust capability for fusing multi-source heterogeneous data (*e.g*., XGBoost's weighted stepwise optimization efficiently captures dynamic trends, as demonstrated by Leigh *et al*. [18] through analyzing the evolution of respiratory support patterns over the first 14 postnatal days). Conversely, while deep learning models (*e.g*., U-Net for image analysis) show promise and align with advances in medical imaging AI (*e.g*., attention mechanisms, volumetric analysis), their overall performance has not consistently surpassed that of ensemble learning, constrained by limitations in interpretability and the demand for large-scale datasets. Notably, the application of model interpretability tools (*e.g*., SHAP values) is maturing, significantly enhancing clinical trust in model decisions [29-31]. For example, Montagna *et al*. [15] identified absent/reversed end-diastolic flow (AREDF) in the umbilical artery as a significant prenatal risk marker (SHAP weight = 0.32), while Lei *et al*. [17] confirmed that the interaction between birth weight <750g and mechanical ventilation >7 days substantially multiplied BPD risk (OR = 9.4). However, translating SHAP-derived feature importance into specific, actionable clinical interventions remains a challenge for integrating these models into practice pathways. The core clinical value of ML models achieving ultra-early prediction within ≤7 postnatal days lies in their potential to transform the traditional BPD management pathway. Current standards require waiting until 36 weeks postmenstrual age for diagnosis, which delays the identification of high-risk infants by 4–8 weeks and misses the critical window for lung development intervention. ML models dramatically shift the risk identification point forward, enabling early targeted interventions. For predicted high-risk infants, the prompt initiation of personalized oxygen therapy (target SpO_2_ 90–94%) becomes feasible, as does consideration of anti-inflammatory treatment (*e.g*., inhaled budesonide) or optimization of lung-protective ventilation strategies. For predicted low-risk infants (negative likelihood ratio LR– = 0.23), unnecessary invasive procedures and drug exposure can be reduced, optimizing healthcare resource allocation. This paradigm shift mirrors the successful clinical pathway of neonatal sepsis risk prediction models (*e.g*., RISK [32]), which utilize early risk stratification to guide precision interventions, ultimately improving clinical outcomes.

However, the substantial heterogeneity observed among studies in this meta-analysis (I^2^ = 78.3%, *p* < 0.001) highlights critical challenges for clinical translation: Data modality disparities and validation bottlenecks: Although multimodal models integrating imaging/biomarkers significantly outperform single-modality (clinical-only) models, the low accessibility of advanced modalities (*e.g*., gastric aspirate Fourier transform infrared spectroscopy, gene expression microarrays) in primary care settings limits model reach. More critically, model performance in external validation (sensitivity 0.75) was markedly lower than in internal validation (0.82), reflecting potential overfitting and limited generalizability. Montagna *et al*. [15] highlighted that the predictive value of umbilical artery Absent/Reversed End-Diastolic Flow (AREDF) was prominent in European cohorts but not significant in Asian cohorts, emphasizing the impact of population differences on model portability. The “gray zone” in BPD diagnosis: Inherent ambiguity exists within diagnostic criteria like NICHD [8], particularly for mild BPD (*e.g*., subjective determination of FiO_2_> 21%), leading to data label noise. Variations in defining “oxygen dependency” across centers further confound the training data foundation. Study design and evidence level limitations: The current evidence is dominated by retrospective cohorts (66.7%), lacking prospective studies that validate the actual clinical benefit of model-guided interventions (*e.g*., whether they demonstrably reduce BPD incidence or save costs). Balancing the prediction time point (≤7 postnatal days) with the optimal intervention window (*e.g*., initiating postnatal corticosteroids within the first week for maximal efficacy) requires models with high specificity (*e.g*., Montagna specificity 0.92 [15]) to ensure intervention precision and avoid overtreatment risks. Cost-effectiveness considerations: Models relying on advanced image analysis (*e.g*., deep learning-based chest X-ray segmentation) or gene expression profiling, while potentially increasing accuracy, also introduce concerns regarding radiation exposure, computational resource consumption, and elevated testing costs (*e.g*., Zhang *et al*. [20]), posing feasibility challenges in resource-limited settings. Furthermore, the use of sensitive genomic/imaging data necessitates careful consideration of data privacy in future multi-center implementations. To achieve effective clinical translation of ML models for BPD prediction, future research must urgently address these key bottlenecks: Promoting standardization and transparency: Mandate adherence to standardized reporting frameworks like transparent reporting of a multivariable prediction model for individual prognosis or diagnosis (TRIPOD) [33-35], requiring comprehensive disclosure of confusion matrices, hyperparameter settings, and feature importance analyses. This addresses the current issue where only 44.4% of studies adequately describe feature selection methods, enhancing reproducibility and comparability. Developing resource-adaptive models: Prioritize the validation and deployment of lightweight models built on routinely available data (*e.g*., vital signs, respiratory rate variability, oxygen saturation trends, basic ventilator parameters) in resource-limited settings, reducing dependence on costly biomarkers and improving accessibility. Enhancing model generalizability and adaptability: Explore strategies for multi-center data integration under privacy-preserving frameworks to boost model universality, and develop adaptable model architectures that allow for parameter fine-tuning based on local population characteristics and clinical practices. Conducting prospective utility validation and health economic evaluation: Design rigorous prospective trials directly comparing BPD incidence, severity, and long-term outcomes between model-guided early intervention groups and standard care groups; perform detailed cost-effectiveness analyses to inform decision-making. Deepening model interpretability and integration strategies: Strengthen the explanation of key variables, result visualization, and design of clinician/patient communication interfaces to improve understanding and adoption. Further expand predictive dimensions and intervention insights by exploring novel early biomarkers related to the immune microenvironment and metabolic signaling, informing future precision prevention and intervention.

## 5 LIMITATIONS

This study has several limitations. First, although this meta-analysis includes 9 studies covering a total of 12,755 preterm infants, the relatively small number of eligible studies and the predominance of retrospective cohort designs (66.7%) inherently limit the strength and generalizability of the evidence. Retrospective studies may be subject to selection bias and information bias, which could impact the robustness of the synthesized conclusions. Second, another important limitation arises from the heterogeneity of BPD diagnostic criteria applied across studies, including variations between the NICHD [8, 36, 37], Jensen grading [38, 39], and NIH consensus definitions. These differences in oxygen dependency thresholds and severity grading introduce inconsistencies that challenge the validity of combined analysis and may affect the interpretation of pooled diagnostic performance. Third, many included studies lack comprehensive documentation of model development procedures, including feature selection methods and hyperparameter tuning. This paucity of technical transparency constrains reproducibility and limits the ability to fully assess the methodological rigor of ML models for BPD prediction. Fourth, restrictions related to medical data privacy have led to a scarcity of open-source code and publicly accessible datasets, hindering independent validation of the findings. Finally, and most critically, the vast majority of the developed models lack prospective validation, leaving their real-world performance yet to be confirmed.

## CONCLUSION

This meta-analysis confirms that machine learning models demonstrate superior performance (AUC = 0.87) for the early prediction of BPD, particularly when integrating multimodal data and temporal dynamic features. Ensemble learning algorithms (*e.g*., XGBoost, Random Forest) consistently demonstrated top performance in most studies, and the inclusion of dynamic respiratory parameters and genetic biomarkers further enhanced predictive accuracy. Current challenges lie in model generalizability and clinical integration, which require resolution through multi-center prospective studies, federated learning frameworks, and standardized reporting guidelines (*e.g*., the TRIPOD-ML statement). Future research must prioritize prospective validation of clinical utility and the development of actionable intervention pathways based on model predictions. Additionally, advancing deep learning methods, particularly those that enhance feature extraction and model robustness, will be crucial. Privacy-preserving multi-center data integration approaches are also crucial for enhancing model generalizability while addressing ethical concerns, thereby further enabling clinical translation. The ultimate goal is to establish a generalizable, reliable, and clinically practical tool for BPD risk stratification to safeguard respiratory health in preterm infants.

## Figures and Tables

**Table 1:** Demographic and clinical characteristics of included studies.

**First Author/Year/Refs.**	**Study Design**	**Country**	**Sample Size**	**GA Range (weeks)**	**BPD Definition**	**Prediction Timepoint**	**Machine Learning Model**	**Key Predictors**	**TP**	**FP**	**FN**	**TN**	**AUC**	**SEN**	**SPE**
Verder (2021) [14]	Multicenter Prospective Cohort	Denmark	61	24–31	NIH consensus (oxygen requirement at 28 days + assessment at 36 weeks PMA)	Birth	SVM + PLS	Gastric fluid FTIR spectroscopy + birth weight + GA + surfactant therapy	23	3	3	32	0.82	0.88	0.91
Montagna (2024) [15]	Retrospective Cohort	Italy	691	<32	NICHD 2018 criteria (respiratory support at 36 weeks + radiological confirmation)	During hospitalization	XGBoost (best model)	Umbilical artery AREDF + GA + mechanical ventilation + ELBW + magnesium sulfate prophylaxis	52	48	33	558	0.92	0.61	0.92
Chou (2024) [16]	Retrospective Cohort	Taiwan, China	1021	22–30	Dual criteria: NICHD (oxygen requirement at 36 weeks)	≤24 hours	U-Net (lung segmentation) + ResNet (BPD prediction)	Imaging features: CXR lung segmentation atlas; Clinical features: postnatal age stratification	347	79	110	485	0.87	0.76	0.86
Lei (2021) [17]	Retrospective Cohort	China	648	24–36	NICHD/NHLBI criteria (respiratory support at 36 weeks)	During hospitalization	Boruta feature selection + Random Forest	Oxygen therapy duration, initial PCO_2_, initial MAP, GA, birth weight, initial FiO_2_	121	50	28	449	0.92	0.81	0.90
Leigh (2022) [18]	Retrospective Cohort	USA	689	≤30.3	2019 Jensen grading (respiratory support mode at 36 weeks, no oxygen concentration)	Postnatal days 1–14	Random Forest + Ensemble Learning	Prenatal factors: GA, sex, race, birth weight Z-score, smoking; Respiratory trajectory: 14-day support mode	276	70	61	282	0.89	0.82	0.80
Khurshid (2021) [19]	Multicenter Retrospective Cohort	Canada	9,006	<33	NIH consensus (oxygen requirement at 28 days + assessment at 36 weeks PMA)	Postnatal days 1/7/14	Penalized Logistic Regression (best model)	GA (categorical), SNAPPE-II score, mechanical ventilation duration, surfactant use, nitric oxide therapy	2710	873	478	4945	0.89	0.85	0.85
Zhang (2025) [20]	Bioinformatics Cohort Analysis	Multinational (GEO)	294	≤32	NIH consensus	Postnatal day 28	LASSO + SVM-RFE + RF (feature selection)	Gene markers: MMP9, Siglec-5, DYSF, MGAM, S100A12; Immune cells: neutrophil, dendritic cell infiltration	50	58	12	174	0.81	0.80	0.75
Dai (2021) [21]	Prospective Cohort	China	245	<32	NIH consensus	Within 7 days postnatal	LASSO Regression (gene-clinical combined model)	Gene markers: BPD-RGS (including OBSL1 and 30 risk genes); Clinical features: GA, birth weight, ventilation	111	23	20	91	0.91	0.85	0.80
Luo (2024) [22]	Bioinformatics Cohort Analysis	China	100	<32	NIH consensus	Postnatal day 28	SVM-RFE + LASSO + RF	Gene markers: CYYR1, GALNT14, OLAH	51	5	11	33	0.89	0.82	0.87

**Abbreviations:** n, number of patients; BPD, bronchopulmonary dysplasia; GA, Gestational Age; PMA, postmenstrual age; NIH, National Institutes of Health; NICHD, National Institute of Child Health and Human Development; NHLBI, National Heart, Lung, and Blood Institute; SVM, Support Vector Machine; PLS, Partial Least Squares; XGBoost, extreme gradient boosting; AREDF, Absent/Reversed End-Diastolic Flow; ELBW, Extremely Low Birth Weight; U-Net, convolutional network for biomedical image segmentation; ResNet, residual neural network; CXR, chest X-ray; MAP, Mean Arterial Pressure; FiO, fraction of inspired oxygen; SNAPPE-II, Score for Neonatal Acute Physiology with Perinatal Extension-II; LASSO, Least Absolute Shrinkage and Selection Operator; SVM-RFE, Support Vector Machine-Recursive Feature Elimination; RF, Random Forest; SHAP, Shapley additive explanations; FTIR, Fourier-Transform Infrared Spectroscopy; BPD-RGS, BPD risk gene signature; AUC, area under the ROC curve; TP, True Positive; FP, False Positive; FN, False Negative; TN, True Negative; SEN, sensitivity; SPE, specificity; GEO, Gene Expression Omnibus; PCO_2_,partial pressure of carbon dioxide.

**Table 2:** Methodological and technical details of machine learning models.

**First Author/Year/Refs.**	**Algorithm Type**	**Input Variable Category**	**Feature Engineering Strategy**	**Validation Strategy**	**Important Variable Contribution Analysis**
**Verder (2021) [** **14** **]**	Support Vector Machine + Partial Least Squares	Multimodal data (Biochemical + Clinical)	PLS dimensionality reduction	Hold-out validation	Regression coefficients
**Montagna (2024) [** **15** **]**	Ensemble learning (XGBoost)	Clinical variables + Prenatal US markers	Recursive feature elimination	5-fold CV + External validation	SHAP values
**Chou (2024) [** **16** **]**	Deep learning (U-Net + ResNet)	Imaging + Clinical data	Convolutional feature extraction	10-fold CV	Class activation mapping
**Lei (2021) [** **17** **]**	Ensemble learning (Random Forest)	Dynamic clinical indicators	Boruta feature selection	Stratified CV	Variable importance
**Leigh (2022) [** **18** **]**	Ensemble learning (Random Forest)	Prenatal factors + Respiratory trajectories	Time-series feature engineering	Time-series CV	SHAP values
**Khurshid (2021) [** **19** **]**	Penalized regression	Clinical variables	Variable clustering analysis	Bootstrap validation	Regression coefficients
**Zhang (2025) [** **20** **]**	Feature selection + Ensemble learning	Genomics + Immune microenvironment	LASSO + SVM-RFE	Independent test set validation	Pathway enrichment analysis
**Dai (2021) [** **21** **]**	Regularized regression	Multi-omics data (Genomics + Clinical)	Gene-clinical feature fusion	5-fold CV	Regression coefficients
**Luo (2024) [** **22** **]**	Feature selection + Ensemble learning	Genomics data	SVM-RFE feature filtering	Hold-out validation	Gene importance score

**Abbreviations:** BPD, bronchopulmonary dysplasia; CV, Cross-Validation; FTIR, Fourier-Transform Infrared Spectroscopy; GA, Gestational Age; LASSO, Least Absolute Shrinkage and Selection Operator; MAP, Mean Arterial Pressure; NICHD, National Institute of Child Health and Human Development; NHLBI, National Heart, Lung, and Blood Institute; NIH, National Institutes of Health; PMA, postmenstrual age; PLS, Partial Least Squares; ResNet, residual neural network; RF, Random Forest; SHAP, Shapley additive explanations; SNAPPE-II, Score for Neonatal Acute Physiology with Perinatal Extension-II; SVM, Support Vector Machine; SVM-RFE, Support Vector Machine-Recursive Feature Elimination; U-Net, convolutional network for biomedical image segmentation; US, ultrasound; XGBoost, extreme gradient boosting.

**Table 3:** Methodological quality assessment of eligible studies: Risk of bias and applicability concerns.

**Study**/Refs.	**Methodological Risk^1^**				**Concerns Regarding Applicability^2^**		
	**Participant Selection**	**Index text**	**Gold Standard**	**Timeline and Progression**	**Participant Selection**	**Index text**	**Gold Standard**
Verder (2021) [14]	L	L	U	L	L	L	L
Montagna (2024) [15]	L	L	U	L	L	L	L
Chou (2024) [16]	L	L	U	L	L	L	L
Lei (2021) [17]	L	L	U	L	L	L	L
Leigh (2022) [18]	L	L	U	L	L	L	L
Khurshid (2021) [19]	L	L	U	L	L	L	L
Zhang (2025) [20]	L	L	U	L	L	L	L
Dai (2021) [21]	L	L	U	L	L	L	L
Luo (2024) [22]	L	L	U	L	L	L	L

**Note:** H = High risk; L = Low risk; U = Unclear. ^1^Methodological Risk Assessment: “Low” risk: All evaluation criteria in a domain receive positive responses.”High” risk: Any negative response to domain criteria (indicating potential bias).”Unclear” risk: Applied when reported data are insufficient for proper evaluation. ^2^Applicability Concerns Evaluation: Follows a parallel structure to bias assessment but excludes signaling questions. Ratings are based on study-relevance alignment: “Low” concern: Strong match with review objectives.”High” concern: Significant mismatch identified.”Unclear” concern: Used when available data cannot support a definitive assessment. The “unclear” rating should only be assigned when inadequate information prevents a reliable judgment in either assessment category.

**Table 4:** Meta-regression analysis of heterogeneity factors in ML-based BPD prediction studies.

**Covariates**	**Multivariable Analysis**			**Sensitivity Estimate (95% CI)**	**Specificity Estimate (95% CI)**
	**LR (Chi square test)**	***p***	**I^2^ index (%)**		
**Algorithm type**	8.32	0.02	76	-	-
Ensemble learning (n=4)	-	-	-	0.84 [0.80-0.88]	0.85 [0.81-0.89]
Single algorithm (n=5)	-	-	-	0.82 [0.78-0.86]	0.83 [0.79-0.87]
**Prediction window**	5.82	0.01	66	-	-
≤7 days postnatal (n=3)	-	-	-	0.83 [0.79-0.86]	0.86 [0.83-0.89]
At 28 days postnatal (n=2)	-	-	-	0.81 [0.78-0.83]	0.81 [0.79-0.84]
**Data modality**	5.82	0.04	66	-	-
Single-modality (n=3)	-	-	-	0.80 [0.77-0.83]	0.80 [0.78-0.82]
Multimodal (n=6)	-	-	-	0.84 [0.80-0.88]	0.85 [0.81-0.89]
**Validation strategy**	8.32	0.02	76	-	-
Internal only (n=6)	-	-	-	0.82 [0.78-0.86]	0.84 [0.80-0.88]
External (n=3)	-	-	-	0.75 [0.68-0.82]	0.80 [0.78-0.84]
**Sample size**	5.82	0.06	66	-	-
N ≥ 500 (n=5)	-	-	-	-	-
N < 500 (n=4)	-	-	-	-	-

**Note:** LR,likelihood ratio; CI, confidence interval; k, number of studies. *p* < 0.01 was considered statistically significant.

## Data Availability

All data generated or analyzed during this study are included in this published article.

## LIST OF ABBREVIATIONS

AUC: Area Under the Curve
AREDF: Absent/Reversed End-Diastolic Flow
BPD: Bronchopulmonary Dysplasia
CI: Confidence Interval
CXR: Chest X-ray
CV: Cross-Validation
DOR: Diagnostic Odds Ratio
ELBW: Extremely Low Birth Weight
FiO_2_: Fraction of Inspired Oxygen
FN: False Negative
FP: False Positive
FTIR: Fourier-Transform Infrared Spectroscopy
GA: Gestational Age
GEO: Gene Expression Omnibus
HSROC: Hierarchical Summary Receiver Operating Characteristic
I^2^: I-squared (Heterogeneity Index)
LASSO: Least Absolute Shrinkage and Selection Operator
LR: Likelihood Ratio
LR+: Positive Likelihood Ratio
LR−: Negative Likelihood Ratio
MAP: Mean Arterial Pressure
ML: Machine Learning
NICHD: National Institute of Child Health and Human Development
NHLBI: National Heart, Lung, and Blood Institute
NIH: National Institutes of Health
PCO_2_: Partial Pressure of Carbon Dioxide
PMA: Postmenstrual Age
PLS: Partial Least Squares
QUADAS-2: Quality Assessment of Diagnostic Accuracy Studies-2
ResNet: Residual Neural Network
RF: Random Forest
ROC: Receiver Operating Characteristic
SEN: Sensitivity
SHAP: Shapley Additive Explanations
SNAPPE-II: Score for Neonatal Acute Physiology with Perinatal Extension-II
SPE: Specificity
SVM: Support Vector Machine
SVM-RFE: Support Vector Machine-Recursive Feature Elimination
TN: True Negative
TP: True Positive
TRIPOD: Transparent Reporting of a Multivariable Prediction Model for Individual Prognosis or Diagnosis
U-Net: Convolutional Network for Biomedical Image Segmentation
US: Ultrasound
VLBW: Very Low Birth Weight
XGBoost: Extreme Gradient Boosting

## STANDARDS OF REPORTING

PRISMA guidelines were followed.

## APPENDIX 1

**Pubmed Search Strategy**:

#1 ("Bronchopulmonary Dysplasia"[Mesh]) OR (((((BPD[Title/Abstract]) OR (Bronchopulmonary Dysplasia[Title/Abstract])) OR (Chronic Lung Disease of Prematurity[Title/Abstract])) OR (Neonatal Chronic Lung Disease[Title/Abstract])).

#2 ("Machine Learning"[Mesh]) OR (((((((Artificial Intelligence[Title/Abstract]) OR (Deep Learning[Title/Abstract])) OR (Neural Networks, Computer[Title/Abstract])) OR (Random Forest[Title/Abstract])) OR (XGBoost[Title/Abstract])) OR (Support Vector Machine[Title/Abstract])) OR (Ensemble Learning[Title/Abstract])).

#3 (((predict*[Title/Abstract]) OR (risk stratif*[Title/Abstract])) OR (prognostic model*[Title/Abstract])) OR (diagnostic model*[Title/Abstract]).

#4 ((("Infant, Premature"[Mesh]) OR (Premature Birth[Mesh])) OR (((((preterm infant*[Title/Abstract]) OR (premature infant*[Title/Abstract])) OR (premature neonate*[Title/Abstract])) OR (VLBW[Title/Abstract])) OR (ELBW[Title/Abstract]))).

#1 AND #2 AND #3 AND #4
